# Silicon as a potential limiting factor for phosphorus availability in paddy soils

**DOI:** 10.1038/s41598-022-20805-4

**Published:** 2022-09-29

**Authors:** Jörg Schaller, Bei Wu, Wulf Amelung, Zhengyi Hu, Mathias Stein, Eva Lehndorff, Martin Obst

**Affiliations:** 1 Leibniz Centre for Agricultural Landscape Research (ZALF), Silicon Biogeochemistry Group, 15374 Müncheberg, Germany; 2 Institute of Bio- and Geosciences, IBG-3: Agrosphere, Forschungszentrum Jülich GmbH, 52428 Jülich, Germany; 3 Institute of Crop Science and Resource Conservation (INRES), Soil Science and Soil Ecology, University of Bonn, Nussallee 13, 53115 Bonn, Germany; 4 Sino-Danish Center for Education and Research, Sino-Danish College, Resource and Environmental College, University of Chinese Academy of Sciences, Beijing, 100049 China; 5 Soil Ecology, University of Bayreuth, Dr.-Hans-Frisch-Str. 1-3, 95448 Bayreuth, Germany; 6 Experimental Biogeochemistry, BayCEER, University of Bayreuth, Dr.-Hans-Frisch-Str. 1-3, 95448 Bayreuth, Germany

**Keywords:** Biogeochemistry, Environmental sciences

## Abstract

Rice cultivation requires high amounts of phosphorus (P). However, significant amounts of P fertilizer additions may be retained by iron (Fe) oxides and are thus unavailable for plants. At the same time, rice cultivation has a high demand for silicic acid (Si), reducing Si availability after short duration of rice cultivation. By studying a paddy chronosequence with rice cultivation up to 2000 years, we show that Si limitation, observed as early as a few decades of rice cultivation, is limiting P availability along the paddy soils chronosequence. Using near edge X-ray absorption fine structure spectroscopy (NEXAFS) in a scanning transmission (soft) X-ray microscope (STXM) we show release of available P was linked to a Si-induced change in speciation of Fe-phases in soil particles and competition of Si with P for binding sites. Hence, low Si availability is limiting P availability in paddy soils. We propose that proper management of Si availability is a promising tool to improve the P supply of paddy plants.

## Introduction

Rice is a major staple food, feeding more than 50% of the world’s population^1^. More than 150 million ha of land are used for rice production^2^. Phosphorus (P) is a main nutrient required for optimal rice plant biomass production and yield^3^. However, in many lowland paddy soils such as in South China, Indonesia, Bangladesh, Pakistan, and India, Farmers have to fertilize huge amounts of P, as the P availability in those soils is low^4–6^. The P binding capacity of the paddy soils increases over time as shown along a paddy chronosequence in the Bay of Hangzhou, China, where rice cultivation up to 2000 years^7,8^ was accompanied by a decreasing P mobility in the soil^8^. Hence, there is the risk that P fertilization is becoming increasingly inefficient with prolonged duration of paddy soil formation.

Beside, P, it is well known that silicon (Si) has positive effect on rice growth^9,10^. Rice production is characterized by a large demand on available Si^11^. Silicon is taken up by the rice root in the form of monosilicic acid and accumulates mainly in the aboveground biomass of the rice plants in form of amorphous silica^12^. Minerals as well as amorphous silica (soluble SiO_2_) can act as a source for different types of silicic acids^13^. Amorphous Si from mineral origin or containing phytolith and diatoms, sponge spicules, radiolarian release more Si compared to mineral^13^. Silicic acid becomes mobilized in form of polysilicic acid which then depolymerizes to monosilicic acid if concentration and pH are both low^13^. Polysilicic acid has a much higher binding strength compared with monosilicic acid^13^. It is found that rice straws contain 300^11^ to 570 kg Si ha^−1^^14^, which is exported from paddy soils upon harvest. As this Si export may outpace the Si inputs from dissolution of soil minerals and addition via irrigation water, rice production may potentially lead to a loss of available (reactive) Si from paddy soil over a few decades^11^. Studies estimating Si availability in soils in China found ~ 20% of the soil being Si deficient and ~ 60% of the soil being very deficient^15,16^. Consequently, paddy soil management including removal of rice straw at harvest will eventually lead to depletion of available and reactive Si in soils^11^.

A loss of reactive Si from paddy soils may impair rice plant performance^17^, but also affect the availability of other nutrients. In particular, Si availability was suggested to be coupled to P availability^18^. This is because silicic acid, eventually mobilized from soil amorphous Si (ASi)^13^, may replace phosphate at the surface of amorphous and crystalline Fe phases, thus increasing available P concentrations in soil solution^19^. Such effect was also shown earlier for monosilicic acid^20^.

At present, it is still unclear to what extent Si availability affects P availability in paddy soils and whether this affect can be related to the time of paddy soil ages. We hypothesized that (1) paddy soils, especially those with long history of rice cultivation, are characterized by low Si availability due to continuous removal of Si by rice harvest, that (2) this low Si availability in paddy soils leads to a decreased P availability due to lower P mobilization by Si competing with P for binding at the surface of soil minerals, and that (3) Si addition increases P availability of paddy soils by releasing P from pedogenic mineral phases such as Fe oxides. To test these hypotheses, we analyzed soil samples of a paddy soil chronosequence (up to 2000 years of rice cultivation) in the Bay of Hangzhou, China^21,22^, regarding the availability of Si and P, as well as the binding and release of P from the surface of particles in paddy soils using synchrotron-based, NEXAFS spectroscopy in a STXM, as this is the only method to distinguish between surface and bulk conditions regarding element speciation.

## Results

### Higher phosphorus availability after soil fertilization by silicon

**Figure 1:**
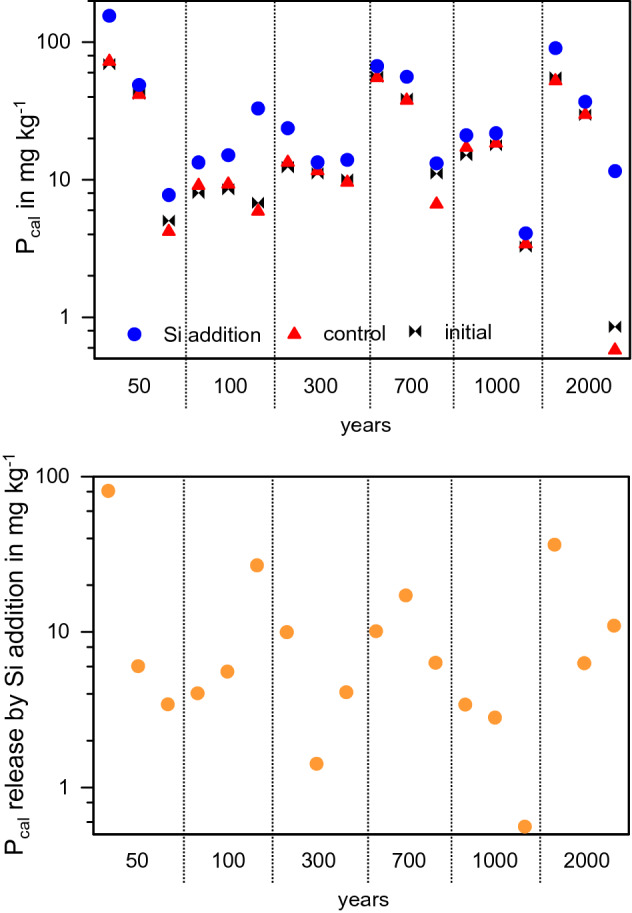
Calcium-acetate-lactate extractable P (P_cal_) prior to (initial) and after incubating paddy soils with different duration of management, and with and without Si addition for two days (upper graph) for soils with different years under paddy managements and different depth (three samples from right to left for each paddy age: ~ 5.7 cm, ~ 14 cm, and ~ 21 cm). The lower graph shows the amount of P released by ASi additions (logarithmic scale; the concentrations of water-soluble P (Figs. S1 and S2) had been added for all samples, respectively).

**Figure 2:**
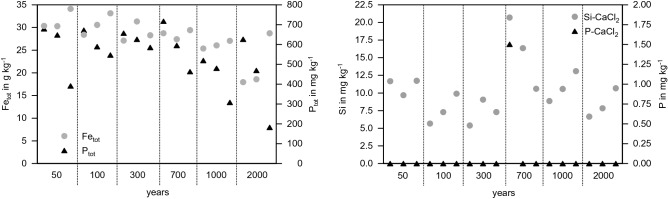
Concentrations of soil total Fe and P (left) and CaCl_2_-extractable Si and P (right) from untreated initial paddy soils.

All samples from the chronosequence of up to 2000 years of rice cultivation showed elevated concentrations of plant-available P (P_cal_) after Si addition (Fig. 1). Overall, ASi-induced increase of P_cal_ ranged from 0.35 and 78 mg P kg^−1^ soil (Fig. 1). The effects were variable for the different Alp, Arp and Ardp horizons, and not consistent across the chronosequence. Nevertheless, the increase in P_cal_ after Si addition (Fig. 1) was smallest in soils with small total P concentrations, and largest in those that were richer in P (Fig. 2, right).

The total P concentrations of those soils ranged from ~ 180 to ~ 720 mg kg^−1^ (Fig. 2, left). The total Fe concentration ranged from ~ 18 to ~ 34 g kg^−1^ (Fig. 2, left). The concentrations of CaCl_2_ extracted P in 17 of the 18 initial paddy topsoils were below the analytical detection limit of the ICP-OES (0.02 mg P L^−1^), and the remaining one had a value of about 1.5 mg kg^−1^ (Fig. 2, left). The CaCl_2_ extractable Si was between ~ 5 and ~ 21 mg Si kg^−1^ with a mean of 10 ± 3.8 mg Si kg^−1^ (Fig. 2, right). Hence, the CaCl_2_ extractable Si as well as P of the initial (untreated) soils was low. The alkaline-extractable ASi concentration in all initial soils was zero (data not shown). Hence, we may consider the paddy soils as both, P and Si limited.

### Changes in iron phases and phosphorus binding after Si addition

**Figure 3:**
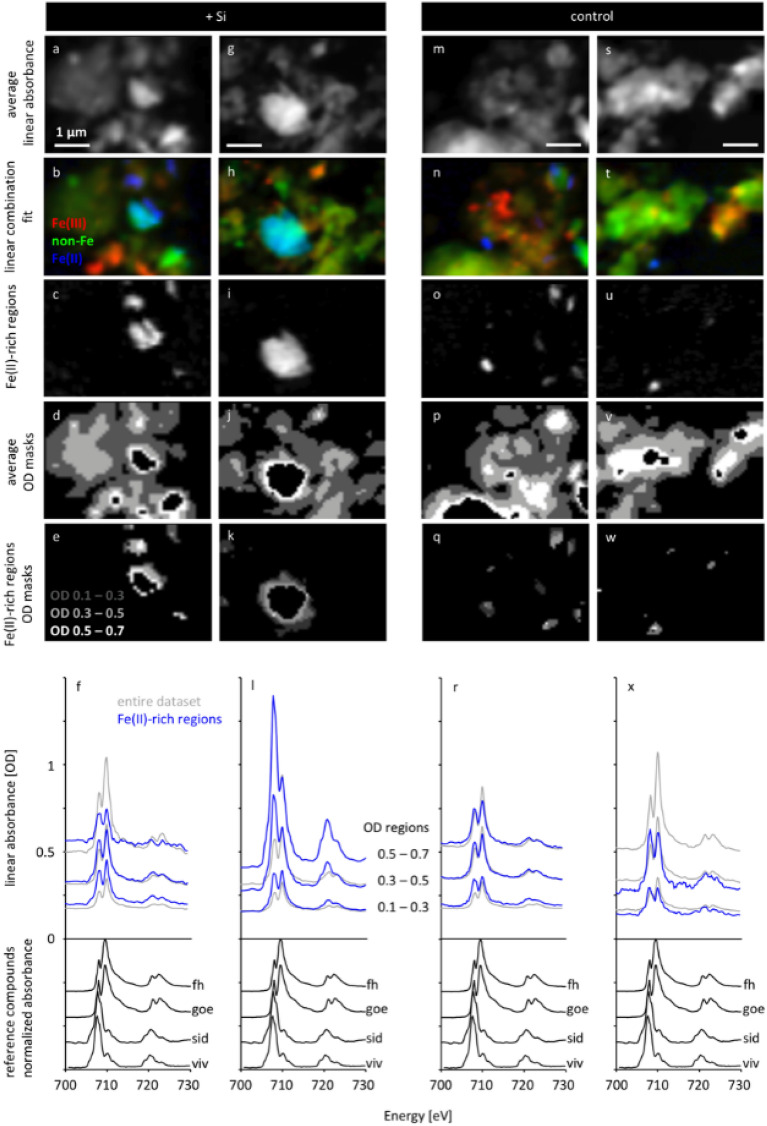
Iron speciation measured soft X-ray NEXAFS at the surface of soil particles showing changes of P binding affected by ASi addition. Four individual soil aggregates were visualized in average OD images (695–760 eV) of 4 × 6 µm^2^ regions of paddy soil samples with (**a**, **g**) and without (**m**, **s**) ASi addition. RGB composite maps of Fe(III)-rich (red), Fe(II)-rich (blue), and non-Fe phases (green) visualize the spatial distribution of the Fe redox states in the soil aggregates of Si-treated (**b**, **h**), and of untreated control samples (**n**, **t**). Specific maps of the Fe(II)-rich phase for Si-treated paddy soil samples (**c**, **i**) and non-treated control samples (**o**, **u**) show the distribution of reduced Fe species (equal to the blue channel in the prior RGB composites). Average OD masks (**d**, **j**, **p**, **v**) based on the entire average OD images (**a**, **g**, **m**, **s**) and OD masks (**e**, **k**, **q**, **w**) based on Fe(II)-rich regions (**c**, **i**, **o**, **u**) were used to extract spectra of regions of increasing thickness. Spectra were extracted using these masks for Si-treated samples (**f**, **l**) and untreated control samples (**r**, **x**), where grey lines represent spectra extracted from the respective entire dataset (**a**, **g**, **m**, **s**), whereas blue lines represent spectra extracted from the Fe(II)-rich regions (**c**, **I**, **o**, **u**) only. Spectra of pure reference compounds ferrihydrite (fh), goethite (goe), siderite (sid), and vivianite (viv) are plotted for comparison. The best linear decomposition fit between the sample and various combinations of reference spectra was achieved with vivanite as the Fe(II) species, suggesting that P is bound to Fe(II) in these soil particles.

To unravel the underlying mechanisms of P mobilization by Si addition, we analyzed Fe speciation and the distribution of the Fe phases using NEXAFS, because Fe phases tend to be the major binding sites for phosphate in soils. While we are aware that the analyses of single soil particles are not necessarily representative of the whole soil^23^, imaging the spatial distribution of all three elements informs on their interactions at microscale^24,25^. The paddy soil with 50-year rice cultivation was selected, since this soil contained the largest concentrations of both the water-soluble P and P_cal_ in the incubation experiment (Figs. 1 and 2). Average linear absorbance, linear combination fit (Fe(II), non Fe and Fe(III), Fe(II)-rich regions, average OD masks, Fe(II)-rich region OD masks, linear absorbance, and reference compounds normalized absorbance were shown for treatments with ASi addition analysis 1 (Fig. 3a–f), treatment with ASi addition analysis 2 (Fig. 3g–l) as well as for control treatment analysis 1 (Fig. 3m–r) and for control treatment analysis 2 (Fig. 3s–x). We found a higher abundance of an Fe(II)-rich phase in the soils after Si addition (blue spectra in Fig. 3l) compared with the control soils (blue spectra in Fig. 3x). Additionally, the Fe(II)-rich phase was redistributed to large agglomerates after Si addition (Fig. 3b,c,h,i,n,o,t,u). The surface of the Fe(II)-rich agglomerates (optical density 0.1–0.3) was different from thick regions (optical density of 0.3–0.5 or even 0.5–0.7). The abundance of Fe(II) decreased towards the surface of the particles (i.e. with decreasing optical density (OD)) for the Si addition treatment. This was most obvious when comparing the ratios of the main absorption peak of Fe(II) at 708 eV and that of Fe(III) at 710 eV (blue spectra in Fig. 3l). This speciation change with thickness was not observed for the control samples (Fig. 3x). We also did not observe this effect when we compared the average spectra of the entire analyzed regions (grey spectra in Fig. 3f,l,r,x respectively).

The Fe(II)-rich regions in our soil sample (blue spectra Fig. 3f,l,r,x) contained both Fe(II) and Fe(III) species. In a linear decomposition, the best fit (i.e. lowest standard deviation) of the sample spectrum was achieved with a combination of goethite (vs. ferrihydrite) and vivianite (vs. siderite) as the respective Fe(III) and Fe(II) phases. Therefore, we interpret the Fe(II)-rich phase as a vivianite-like Fe(II)-phosphate phase of low crystallinity.

Additionally to this, we analyzed the soil pH and zeta potential for the different soil after incubation for control and Si addition treatment. However, we found no clear pattern for zeta potential with positive and negative values of delta zeta potential (difference between zeta potential for control and Si addition treatment) (Table S1). For the soil pH after incubation, we found also no clear patter as the difference in soil pH between control and Si addition treatment was between 0 and − 0.5 (Table S1).

## Discussion

Rice plants are well known for their very high Si accumulation in the aboveground biomass^12^. Due to the removal of aboveground biomass by harvest, rice paddy soils are prone to desilication leading to low Si availability in the paddy soils^11^. Here we show that ASi addition is increasing P availability in all studied paddy soils along a 2000 years chronosquence.

The Si availability in all soils of the studied chronosequence was low (most soils below 10 mg Si kg^−1^) compared with other paddy soils, which contain available Si with a mean concentration of ~ 30 mg kg^−1^ depending on soil pH and maximum values of > 80 mg kg^−1^^26^. This is in line with other studies showing low Si availability in paddy soils^15,16^. This low Si availability may even be lower during the main phase of growing season as the rate of Si uptake by rice is faster as that of dissolution of Si-containing soil minerals^27^. Dissolved Si is in the form of polysilicic acid and equilibrates with monosilicic acid in soil solution depending on pH and concentration^13^. The lower the Si concentration in the soil solution is, the more the silicic acid speciation shifts from polysilcic acid (with high binding affinity to soil minerals) towards monosilicic acid (with low binding affinity to soil minerals)^13^. Hence, at low Si concentration in soil solution, little Si competes with P for binding to soil minerals^13^, i.e., potentially little if any P may be replaced by Si from mineral binding sites, as most Si in solution is suggested to be monosilicic acid^13^ and monosilicic acid is suggested to be able to mobilize only low amounts of P, as P has a much higher binding affinity compared to monosilicic acid^20^. However, earlier studies showed that monosilicic acid is not able to compete with phosphate for binding sites at the surface of pure minerals^28^. However, our data does not confirm this earlier findings as in our experiment soil was used insteed of pure minerals and ASi producing polysilicic acid with different binding affinity to monosilicic acid.

The concentrations of available P in the studied paddy soils were low. However, after Si addition, the concentration of available P increased to values above the critical value of P availability for rice cultivation of about 3.5 mg P kg^−1^. Furthermore, Si addition increased the Si concentration in soil solution (Fig. S2) and potentially shifted the Si speciation in soil solution toward polysilicic acid^13^. This increase in Si concentration in soil solution and the potential shift toward polysilicic acid (based on higher Si concentrations in soil solution) led to P mobilization from soil particles^29^.

The biogeochemistry of paddy soils is particularly dominated by redox sensitive elements such as Fe^21^. Repeated flooding of paddy soil repeatedly induces the reduction of Fe(III) and releases Fe(II) from soil minerals, reducing the binding capacity of soil for P, and promoting a mobilization of P into the liquid phase. The process goes along with a reduction of Fe(III) phases and potential formation of new Fe(II) minerals, such as vivianite (Fe^2+^_3_(PO_4_)_2_·8H_2_O). It usually binds P less strongly than Fe(III) oxidic phases like ferrihydrite, goethite, lepidocrocite or hematite^30^.

High Si availability seems to increase the fraction of Fe(II) compared to Fe(III) (compare blue spectra Fig. 3l,x). Apart from natural soil heterogeneity at microscale^23^, a higher share of Fe(II) phases may be explained by the fact that the formation of silica gel from ASi^31^ favors reducing conditions^32,33^, as ASi reduces the hydraulic conductivity at low matric potential and with this potentially reducing the transport of electron acceptors. Silicon also hinders the transformation of poorly crystalline minerals to more stable crystalline Fe(III) phases^34^. By contrast, desilication of paddy soils can change the Fe mineralogy to more crystalline Fe(III) phases over time. As desilication of paddy soil can take place in less than 50 years of rice cultivation, the change in Fe mineralogy and related P dynamics can already happen within a few decades.

The mobilized P and Fe partly formed new particles of small sizes (tens of nanometers). The NEXAFS measurements showed that relatively larger and Fe(II)-rich particles are formed in the Si addition compared to the control treatment (Fig. 3l,x), which can be interpreted as a vivianite-like Fe(II)-phosphate phase^35^. Subsequently, the mobilized P is coprecipitated in Fe(II) mineral phases as Fe(II)-P (e.g. vivianite)^36^. As the Fe–P binding in Fe(II)-phosphates is weaker than in Fe(III)-phosphates^30^, Fe(II)-phosphates have higher potential of re-releasing P. Consequently, the process of vivianite formation by Si addition further contributes to improved P availability. The improved P mobilization by increasing Si availability in Si-depleted soil can thus be taken into consideration as potential option to improve P use efficiency in paddy soils and therewith to reduce P fertilizer requirements. Future studies should analyze plant P status to quantify to which degree Si addition also improves P nutrition.

In this study, we showed that low Si availability limits P availability in the soils of a paddy chronosequence. We attribute this finding to the fact that polysilicic acid released at high Si concentrations outcompeting P at Fe(III)-containing binding sites and that the addition of ASi, favoring reducing conditions, leads to a transformation of Fe(III) to Fe(II), so that P is either directly released into soil solution or bond less strongly to Fe(II)-containing minerals like vivianite. To increase the Si availability and the ASi amounts of paddy soils, farmers can take advantage of approaches that recycle rice straw residuals, such as rice straw burning^37^ or accelerated composting^38^, in order to replenish plant-available Si in soil as shown for other crops^39,40^, or use different Si fertilizers^41^. Optimizing Si fertilization requirements for co-beneficial needs of P fertilization in paddy soils may now warrant further attention. And finally, desilication of paddy soil eventually leads to decreased P availability and higher demand for P fertilizer in paddy soils.

## Material and methods

### Sampling of paddy soils

Earlier works by Cheng et al.^7^ and, subsequently, in further detail by Kölbl et al.^21^ had identified a chronosequence of soils with 0–2000 years under paddy management. Site selection had been based on the construction ages of dykes, that started 2000 years ago in the region near Cixi, and that moved towards the sea with increased population growth. After dyke construction, paddy management started on former wetland soils with Yangtze river sediments as parent materials. Here, we analyzed stored samples taken by Roth et al.^22^. The traditional paddy management practice in this region is a crop rotation of rice in the wet season followed by wheat or other upland crops in the dry season^42^. Annual precipitation averages 1325 mm with a maximum between April and October. The mean annual temperature is 16.3 °C^7^.

In spring 2008, after the harvest of upland crops and at the time of rice transplanting, a chronosequence of six soils being 50, 100, 300, 700, 1000 and 2000 years under paddy managements, was sampled by horizon to a depth of > 100 cm. Here we analyzed topsoil only, i.e., from 0 to approximately 20 cm (Alp, Arp and Ardp horizons, for complete soil description, see^21^). At all sites, three field replicates were sampled. The age of the sites was reconstructed from the historical records of the time of dyke construction bordering these areas^22^.

Soil texture ranged from silty to clayey loam. The paddy profiles were generally characterized by a puddled layer, low in bulk density (~ 1 g cm^–3^) and an underlying dense (1.4–1.6 g cm^–3^) plough pan (Ardp horizon). Horizons below were differentiated by increasing mottling with Fe and manganese concretions. The younger paddy soils (50-, 100-, 300-year old) were classified as Anthraquic Cambisols, and older paddy soils (700-, 1000-, 2000-year old) were classified as Hydragric Anthrosols (WRB, 2007) due to prolonged flooding-drainage cycles and variation of redox conditions also in subsoils^21^. All samples were air-dried, sieved to a size < 2 mm prior to chemical analyses.

### Experimental setup of the incubation experiment

At each site, several kg soil were sampled from different subsites^21^. Here, aliquots of 3.5 g of the paddy soil from each site of the chronosequence were mixed with ASi (amorphous silica, Aerosil 300, Evonik Industries, Germany) to achieve an ASi addition of 3%. And then 30 mL pure water was added to allow the ASi to react with the soil and release silicic acid. For the control treatment, only 30 mL pure water was added to the same amount of paddy soil. ASi is a hydrophilic fumed silica with a surface area of ~ 300 m^2^ g^−1^. As the ASi is only weakly buffered regarding pH, the addition of ASi had no effect on soil pH (data not shown). All samples were put on a rotary shaker (30 rpm) for 48 h to ensure the reaction of the ASi with the soils. As the samples contained real soils and not selected minerals as in controlled but artificial lab studies^43^, we unfortunately had to refrain from using theoretical modelling to describe defined sorption mechanisms or energies.

### Element extractions and analyses

Total P and Fe content (Fe_tot_, P_tot_) of the paddy soils were analysed by an inductively coupled plasma—optical emission spectrometer (ICP-OES) after *aqua regia* extraction in a microwave digestion system (Mars5, CEM, Germany)^44^. A Chinese standard reference material GBW7604 (Office of Certified Reference Material, Langfang, China) was extracted and analysed together with the soil samples for the validation of the analytical procedures of total concentration determination. CaCl_2_ extractable Si and P from the paddy soils were extracted by using 0.01 M CaCl_2_ for 24 h. The alkaline-extractable amorphous Si (ASi) was extracted by using 0.1 M NaCO_3_ for 1, 3, and 5 h to determine the ASi concentration by plotting the extracted amounts against the extraction time and the y-axis intercept of the linear least-squares regression line represents the estimated ASi content^45,46^. As the soils of the incubation experiments contained water, which had already mobilized a similar amount of P to that extracted by 0.01 M CaCl_2_, we applied a calcium-acetate-lactate extraction to quantify available P (P_cal_) after the incubation experiment, and also for initial samples according to Schüller^47^. The data of P availability (P_cal_) in the different soils (Fig. S1) were corrected by of the amount of P mobilized (water soluble P) during incubation, i.e. water soluble P was added to P_cal_ (Fig. S2). All extractions and analyses were done in duplicates. The Si and P concentrations of respective extracts were analyzed by ICP-OES.

Soil pH values of the samples after incubation were measured in deionized water at a solid to solution ration of 1:2.5. 1 g of the samples was suspended in 2.5 ml of deionized water and equilibrated for 60 min before measurement. For zeta potential measurement after incubation, 10 mg of the soil were suspended in 40 ml of pure water and mixed on a Vortex mixer for 2 min. One ml of the suspension was transferred into disposable zeta potential cuvettes. Zeta potential was measured by phase analysis light scattering (PALS) using a Zetasizer Nano ZSP (Malvern Panalytical, Herrenberg, Germany) equipped with a He–Ne laser (λ = 633 nm) and a non-invasive backscatter detector at a fixed angle of 173°.

### Spatially resolved NEXAFS analysis

The soil suspensions of the incubation experiments were centrifuged at 2370×*g* for 5 min to sediment large organic particles that are not X-ray transparent for the subsequent analysis by synchrotron-based scanning transmission (soft) X-ray microscopy (STXM). Five microliter of the supernatant were wet-deposited onto formvar-coated Cu grids (300 mesh, Plano GmbH, Wetzlar, Germany), blotted immediately, air dried and stored in dry atmosphere until analysis. The samples were analyzed by STXM at the spectromicroscopy beamline 10ID-1 at the Canadian Light Source (CLS), Saskatoon SK, Canada^48^. The beamline was operated at an energy resolving power E/∆E P 3000. The samples were analyzed in 1/5 of an atmosphere of He. Image stacks of sample regions of 6 µm × 4 µm were recorded across the Fe2p absorption edges (699–740 eV), with a pixel size of 80 × 80 nm^2^, and with an energy resolution of 0.15 eV in the energy region of interest. The datasets were analyzed using the aXis2000 software package^49^. Spectra were extracted from distinct regions dependent on the average linear absorbance of the particles across the absorption edge. This provides information on the Fe speciation in dependence of the distance from the surface of the particles and thus allows for identifying surface-related speciation differences. As the average cumulative thickness of the respective Fe mineral phase in the (sub-) micron sized soil aggregates was as low as few nm, only the Fe L_3,2_ edges with the high resonances provide sufficient sensitivity to allow for identifying surface-related binding speciation changes at relevant sub-µm spatial scales. Unfortunately both Si 1 s and P1s absorption edges (1839 and 2154 eV respectively) do not nearly provide the required absorption (or fluorescence) intensities for spatially resolved spectral studies of particles in this size range, so that only the detection of P in Fe-mineral phases was possible in this study.

The image stacks were aligned and converted from transmission into linear absorbance scale (denoted as optical density, OD) according to the formula OD = − ln(I/I_0_) where I represents the measured photon intensity at each individual pixel and I_0_ represents the incoming photon intensity measured in an empty area adjacent to the regions of interest.

Fe(II)- and Fe(III)-rich regions of the datasets were mapped by linear spectral decomposition of the image stacks^50^ using spectra of Fe(II)-, Fe(III)-rich regions previously extracted from the dataset. Additionally, a modelled non-Fe spectral background absorption, based on the atomic scattering factors^51^, was used for linear spectral decomposition, assuming a composition of organic carbon (CH_2_O). Iteratively, spectra of the Fe(II)- and Fe(III)-rich regions were then extracted by using threshold masks derived from previously calculated species maps. Based on the OD ranges OD 0.1–0.3, OD 0.3–0.5 and OD 0.5–0.7 of the average absorption image across the Fe2p edges, spectra were then extracted from both the entire dataset and from the previously mapped Fe(II)-rich region only. Thicker regions were not considered to avoid problems with absorption saturation^52^.

## Supplementary Information

Supplementary Information.

## Data Availability

The datasets used and/or analyzed during the current study is available from the corresponding author on reasonable request.
