# Supplementary material for: Silicon as a potential limiting factor for phosphorus availability in paddy soils
Source: Sci Rep. 2022 Sep 29;12:16329. doi: 10.1038/s41598-022-20805-4 (PMC9521874; doi:10.1038/s41598-022-20805-4)
Supplement: Supplementary file 1 — Supplementary Information. [file 41598_2022_20805_MOESM1_ESM.docx]

**Supplementary information to:**

Silicon as potential limiting factor for phosphorus availability in paddy soils

Jörg Schaller ^1, *^, Bei Wu ^2^, Wulf Amelung ^3^, Zhengyi Hu ^4^, Mathias Stein ^1^, Eva Lehndorff ^5^, and Martin Obst ^6^

^1^ Leibniz Centre for Agricultural Landscape Research (ZALF), Silicon biogeochemistry group, 15374 Müncheberg, Germany

^2^ Institute of Bio- and Geosciences, IBG-3: Agrosphere, Forschungszentrum Jülich GmbH, 52428 Jülich, Germany

^3^ Institute of Crop Science and Resource Conservation (INRES), Soil Science and Soil Ecology, University of Bonn, Nussallee 13, 53115 Bonn, Germany

^4^ Sino-Danish Center for Education and Research, Sino-Danish College, Resource and Environmental College, University of Chinese Academy of Sciences, Beijing 100049, China.

^5^ Soil Ecology, University of Bayreuth, Dr.-Hans-Frisch-Str. 1-3, Bayreuth 95448, Germany

^6^ Experimental Biogeochemistry, BayCEER, University of Bayreuth, Dr.-Hans-Frisch-Str. 1-3, 95448 Bayreuth, Germany

The supporting information contains two figures (Figs. S1 and S2) and on table (Table S1).

Figure S1 Calcium acetate lactate extractable P (P_cal_) prior (initial) and after the incubation experiment of the different soil with and without Si addition for two days (upper) for soils with different years under paddy managements and different depth (three samples for each paddy age from right to left each: ~5.7 cm, ~14 cm, and ~21 cm) and the differences between Si-treated and non-treated soil for P_cal_ (lower), shown on a logarithmic scale.

Figure S2 Soluble Si (upper), P (middle) for control and Si addition treatment as well as the differences in P mobilization between Si addition and control treatment (lower) for soils with different years under paddy managements and different depth (three samples for each paddy age from right to left each: ~5.7 cm, ~14 cm, and ~21 cm).

Table S1: Zeta potential and soil pH after the incubation experiment for the Si addition and the control treatment and the differences between Si addition and control treatments.

|  | Zeta potential (mV) | |  | pH | pH |  |
| --- | --- | --- | --- | --- | --- | --- |
| years | control | Si | delta Zeta | control | Si | delta pH |
| 50 | -14,70 | -20,33 | -5,63 | 7,81 | 7,62 | -0,2 |
| 50 | -17,37 | -20,83 | -3,47 | 8,10 | 8,13 | 0,0 |
| 50 | -16,80 | -14,80 | 2,00 | 8,38 | 8,12 | -0,3 |
| 100 | -21,27 | -28,50 | -7,23 | 5,94 | 5,85 | -0,1 |
| 100 | -26,73 | -26,07 | 0,67 | 6,79 | 6,4 | -0,4 |
| 100 | -24,30 | -20,60 | 3,70 | 7,91 | 7,41 | -0,5 |
| 300 | -16,35 | -28,83 | -12,48 | 6,42 | 6,09 | -0,3 |
| 300 | -22,47 | -26,37 | -3,90 | 6,70 | 6,56 | -0,1 |
| 300 | -17,37 | -24,67 | -7,30 | 8,45 | 8,23 | -0,2 |
| 700 | -22,70 | -27,97 | -5,27 | 7,36 | 7,15 | -0,2 |
| 700 | -26,30 | -20,20 | 6,10 | 6,95 | 7,05 | 0,1 |
| 700 | -24,73 | -24,70 | 0,03 | 7,35 | 7,01 | -0,3 |
| 1000 | -26,40 | -20,53 | 5,87 | 6,40 | 6,3 | -0,1 |
| 1000 | -26,03 | -26,53 | -0,50 | 6,98 | 6,64 | -0,3 |
| 1000 | -25,37 | -24,87 | 0,50 | 7,63 | 7,24 | -0,4 |
| 2000 | -23,20 | -27,83 | -4,63 | 6,08 | 5,86 | -0,2 |
| 2000 | -17,77 | -25,50 | -7,73 | 5,98 | 5,78 | -0,2 |
| 2000 | -22,93 | -23,97 | -1,03 | 7,28 | 6,97 | -0,3 |
